# Incidence of radiodermatitis in breast cancer patients during hypofractionated radiotherapy

**DOI:** 10.1590/1980-220X-REEUSP-2022-0173en

**Published:** 2022-12-05

**Authors:** Larissa Aparecida Corrêa Vieira, Amanda Gomes de Menêses, Priscila de Sousa Maggi Bontempo, Giovana Paula Rezende Simino, Elaine Barros Ferreira, Eliete Neves da Silva Guerra, Paula Elaine Diniz dos Reis

**Affiliations:** 1 Universidade de Brasília, Faculdade de Ciências da Saúde, Laboratório Interdisciplinar de Pesquisa Aplicada à Prática Clínica em Oncologia, Brasília, DF, Brazil.; 2 Universidade Federal de Minas Gerais, Belo Horizonte, MG, Brazil.; 3 Universidade de Brasília, Faculdade de Ciências da Saúde, Laboratório de Histopatologia Oral, Brasília, DF, Brazil.

**Keywords:** Radiodermatiti, Radiotherap, Breast Neoplasm, Oncology Nursin, Radiation Dose Hypofractionation, Radiodermatitis, Radioterapia, Neoplasias de la Mama, Enfermería Oncológica, Hipofraccionamiento de la Dosis de Radiación, Radiodermatite, Radioterapia, Neoplasias de Mama, Enfermagem Oncológica, Hipofracionamento da Dose de Radiação

## Abstract

**Objective:**

To analyze the incidence, dose of occurrence, grade, severity, and associated risk factors for the development of radiodermatitis, by area of the irradiated breast, in women with breast cancer, during hypofractionated radiotherapy.

**Method:**

Observational, prospective, and longitudinal study, according to the guidelines of the Strengthening the Reporting of Observational studies in Epidemiology, carried out between May 2019 and May 2021.

**Results:**

A total of 104 women participated in the study, and 73.1% (95%CI: 64–82) developed signs of radiodermatitis during treatment. The majority (63.5%, 95%CI: 54–73) developed erythema in the axillary region with about 36.5 Grays. Women with large breasts and statin users are more likely to develop radiodermatitis. However, women with Phototype III skin color classification (light brown skin) are less likely to develop radiodermatitis, with skin color being a protective factor.

**Conclusion:**

The incidence of radiodermatitis in women with breast cancer during hypofractionated radiotherapy is significant. Therefore, the development of protocols for the management of this radiotoxicity is suggested, considering the cumulative dose and associated risk factors.

## RESUMO

**Objetivo:**

Analisar a incidência, a dose de ocorrência, o grau, a severidade e os fatores de risco associados para o desenvolvimento de radiodermatite, por área da mama irradiada, em mulheres com câncer de mama, durante a radioterapia hipofracionada.

**Método:**

Estudo observacional, prospectivo e longitudinal, conforme diretrizes do Strengthening the Reporting of Observational studies in Epidemiology, realizado entre maio de 2019 e maio de 2021.

**Resultados:**

Participaram do estudo 104 mulheres, 73,1% (IC95%: 64–82) desenvolveram sinais de radiodermatite durante o tratamento. A maioria (63,5%, IC95%: 54–73) desenvolveu eritema na região axilar com cerca de 36,5 Grays. Mulheres com mamas volumosas e usuárias de estatinas possuem maior chance de desenvolver radiodermatite. Entretanto, mulheres com a cor da pele classificada como Fototipo III (pele morena clara) possuem menor chance de desenvolver radiodermatite, sendo a cor da pele um fator protetor.

**Conclusão:**

A incidência de radiodermatite em mulheres com câncer de mama durante a radioterapia hipofracionada é expressiva. Sugere-se, portanto, o desenvolvimento de protocolos para o manejo desta radiotoxicidade, considerando a dose cumulativa e fatores de risco associados.

## RESUMEN

**Objectivo:**

Analizar la incidencia, dosis de ocurrencia, grado, severidad y factores de riesgo asociados para el desarrollo de radiodermatitis, por área de la mama irradiada, en mujeres con cáncer de mama, durante radioterapia hipofraccionada.

**Método:**

Estudio observacional, prospectivo y longitudinal, según las directrices del Strengthening the Reporting of Observational studies in Epidemiology, realizado entre mayo de 2019 y mayo de 2021.

**Resultados:**

Participaron del estudio 104 mujeres, el 73,1% (IC 95%: 64–82) desarrollaron signos de radiodermatitis durante el tratamiento. La mayoría (63,5%, IC95%: 54–73) desarrolló eritema en la región axilar con alrededor de 36,5 Grays. Las mujeres con senos grandes y usuarias de estatinas tienen más probabilidades de desarrollar radiodermatitis. Sin embargo, las mujeres con color de piel clasificado como Fototipo III (piel morena clara) tienen menos probabilidades de desarrollar radiodermatitis, siendo el color de piel un factor protector.

**Conclusión:**

La incidencia de radiodermatitis en mujeres con cáncer de mama durante la radioterapia hipofraccionada es significativa. Por lo tanto, se sugiere el desarrollo de protocolos para el manejo de esta radiotoxicidad, considerando la dosis acumulada y los factores de riesgo asociados.

## INTRODUCTION

Breast cancer is the leading cause of cancer death in women worldwide, accounting for 11.7% of all cases and 6.9% of deaths^(1)^. Treatment for breast cancer is multimodal and about 50% of patients will undergo radiotherapy at some stage of treatment planning^(1)^. Radiotherapy is effective in tumor control and consists of the use of X-rays with high-intensity wavelengths (photons) and other forms of ionizing radiation to treat cancer. Ionizing radiation is capable of causing damage to the genetic material of cells, directly or indirectly affecting their structure and composition^(3,4)^. Conventional radiotherapy protocols for breast cancer recommend a daily application schedule of 1.8 to 2.0 Grays (Gy), lasting 5 to 6 weeks. On the other hand, hypofractionated radiotherapy (HFRT) protocols adopt application above 2.02 Gy to 4.0 Gy per fraction, and have a duration of 3 to 4 weeks^(5)^.

Studies comparing HFRT with conventional radiotherapy showed equivalent rates of overall survival and local tumor control between the two treatment regimens, but encouraged the use of HFRT in women with early breast cancer, as they provide greater efficiency in the use of resources, although the results have been shown to be equivalent in terms of efficacy, toxicity, and cost-effectiveness^(3,6)^. RTHF reduces treatment time and, consequently, waiting lines, providing lower cost per patient for the service, as well as benefiting patients whose distance between their residence and the service and treatment time can be obstacles^(7,8)^. However, in both radiotherapy protocols, ionizing radiation directly damages the skin and deep tissue cells, causing dryness, loss of elasticity, pigmentation, fibrosis, capillary dilatation and radiodermatitis^(9)^.

Radiodermatitis can manifest acutely or chronically. Acute radiodermatitis occurs within hours to weeks after starting radiotherapy, with an incidence of 98% in breast cancer patients^(10)^. It manifests as skin discoloration, such as erythema or hyperpigmentation, edema, epilation, moist and dry desquamation^(3,9)^. Reaction intensity is dose-dependent, that is, it is directly associated with the accumulation of ionizing radiation dose in the skin during the treatment. In addition, intrinsic and extrinsic factors such as Body Mass Index (BMI), breast size, age, sex, sun exposure, smoking and drinking history, concomitant hormone treatment and planning techniques can interfere with the occurrence and severity of the reaction^(3,9,11)^. A study highlighted that skinfolds can be sources of friction and moisture, which increases the chance of skin lesions, being commonly observed in obese patients with large breasts, especially in the inframammary fold region^(11)^. As a consequence, radiodermatitis has a direct impact on the patient’s quality of life and, in more severe levels, can limit the prescribed therapeutic dose, lead to treatment interruption, and potentially compromise local disease control and survival rate^(3,4)^.

Although the pathophysiology of radiodermatitis is known, there is a gap regarding the incidence and distribution of the grading of this radiotoxicity throughout the treatment, considering the dose and the area of the breast irradiated in patients undergoing HFRT. Studies with these data can contribute to the elaboration of recommendations and the standardization of protocols for radiodermatitis prevention and management in a more specific way. Thus, this study aimed to analyze the incidence, dose of occurrence, level, severity, and associated risk factors for the development of radiodermatitis, per area of the irradiated breast, in women with breast cancer during hypofractionated radiotherapy.

## METHODS

### Study Design, Period and Local

Observational, prospective, longitudinal study, reported according to the guidelines of the *Strengthening the Reporting of Observational Studies in Epidemiology* (STROBE)^(12)^. Data collection was carried out at the Radiotherapy Outpatient Clinic of the High Complexity Oncology Unit of the University Hospital of Brasília (UNACON/HUB) from May 2019 to May 2021.

### Population

Women aged 18 years or older diagnosed with breast cancer undergoing HFRT treatment.

### Inclusion and Exclusion Criteria

The sample consisted of breast cancer patients who underwent HFRT. Patients aged 18 years or older, diagnosed with breast cancer at any clinical stage, with an indication for HFRT were included. Exclusion criteria were: indication of urgent radiotherapy due to hemorrhage in the irradiated breast area and patients with a previous history of radiotherapy in the same irradiated field.

### Data Collection

Patient recruitment took place from May 2019 to May 2021 during the first nursing consultation held on the first day of radiotherapy. Following provision of information about the procedures related to the day, time, number of sessions and doses that would be applied, adverse effects and necessary care during treatment, the patient was invited to participate in the study, receiving the necessary information regarding the research. If there was agreement to participate, the Free and Informed Consent Form (FICF) and the Image Authorization Form for research purposes were signed.

Information regarding care during treatment was delivered through a validated educational manual, routinely used in the department, highlighting skin care, clothing, and hygiene^(13)^. Regarding the use of products on the skin, all patients received a liposomal gel to apply on the irradiated area, as recommended by the service.

All patients were treated in radiotherapy devices, of the Linear Accelerators type, manufacturer VARIAN^®^, model CLINAC CX or manufacturer SIEMENS^®^, model PRIMUS, using three-dimensional conformational planning (3D – CRT), with therapeutic protocols ranging from 15 to 20 sessions.

During the first nursing consultation, patients’ sociodemographic and clinical data were recorded using an instrument prepared by the authors. The data collected were: age (in years, with the date of the first consultation as a time frame), sex, education, skin phototype (Fitzpatrick’s phototype classification)^(14)^, smoking and drinking status (never, stopped for more than six months or less than six months and currently), skin disease (yes/no), diabetes (yes/no), hypothyroidism (yes/no), statin use (yes/no), current medications, BMI, chest circumference, diagnosis, tumor staging, histopathology, concomitant treatment with hormone therapy (yes/no), irradiated breast, irradiated region, planning technique, predicted fractions, cumulative total dose of radiation (in Gy), fractional dose (in Gy) and energy used.

Skin assessment was performed daily following the criteria of the “Acute Radiodermatitis Grade” (ARDG) scale: 0 (no change), 1a (hyperpigmentation or mild erythema), 1b (intense erythema), 2a (skin dryness), 2b (localized dry desquamation at one or more separate points), 2c (dry desquamation disseminated in one or more contiguous points), 3a (moist desquamation in folds), 3b (moist disseminated desquamation), 4 (Bleeding and/or ulceration) and 5 (Necrosis)^(15)^. Symptoms reported by patients during treatment were recorded weekly, namely: “Local heat”, “Burning”, “Itching”, “Report of rough, dry and/or tight skin” and “Pain”^(15)^.

Skin assessments were performed daily until the end of HFRT and photos were taken weekly. After the patient developed some degree of radiodermatitis, the photos were made on alternate days. For the photographic record from the mammary region a camera of the Smartphone Asus Zenfone Max Shot 64 GB, Full HD Plus 6.2” Screen, Octa Core, Triple Camera 12 MP + 5 MP + 8 MP. The records were made in a standardized way respecting the same conditions of lighting, distance, and positioning. The patients’ identities were preserved.

### Data Analysis and Treatment

For data analysis, descriptive and inferential statistics were used with calculation of mean and standard deviation (SD). For factors associated with a greater chance of developing radiodermatitis, a multivariate analysis was performed and the results were presented as Odds Ratio (OR). Confidence intervals were established for the occurrence of each degree of radiodermatitis, considering a significance index of 5%, using the software *Statistical Package for Social Science* (SPSS), version 22.0.

### Ethical Aspects

The study was approved by the Research Ethics Committee (*CEP*) of the Health Sciences School of the Universidade de Brasília (FS/UnB), number 3.123.117, approved in 2019, and in compliance with Resolution 466/12. The inclusion of patients took place through the signature of the informed consent and the Image Authorization Term for research purposes.

## RESULTS

A total of 104 women were included in the study, aged between 31 and 86 years (average of 52.6), with completed high school being the most frequent level of education (30.8%). Regarding skin characteristics, 56 participants (53.8%) had phototype III (light brown skin, with normal sensitivity to the sun and moderate burning) and 99 (95.2%) did not have any skin disease. As for smoking and alcohol consumption, 73 participants (70.2%) never used tobacco and 66 (63.5%) never consumed alcoholic beverages. Regarding health status, 88 participants (84.6%) did not have diabetes, 102 (98.1%) did not have hypothyroidism, 88 (84.6%) did not use statins, 41 (39.4%) had a BMI greater than 25 or less than 29.9 (mean = 28.5; SD = 5.4), characterizing overweight according to the World Health Organization (WHO), and 61 (58.7%) had voluminous breast, with a chest circumference greater than 98 cm, corresponding to the “L” bra size (mean = 101.1; SD = 11.8). The sociodemographic and clinical characterization of the sample is available in Table 1. There were no follow-up losses, but it should be noted that 49 patients had from 1 (n = 27) to 2 (n = 22) days of delay due to treatment interruption due to linear accelerator maintenance.

**Table 1.** Sociodemographic and clinical characterization of the sample (n = 104). Brazil, 2021.

Characteristics	n = 104
**Age in years,** Mean (SD)	52.6 (12.2)
**Chest circumference in cm,** Mean (SD)	101.1 (11.8)
**Sex**, n (%)	
Female	104 (100)
**Phototype**, n (%)	
II	14 (13.5)
III	56 (53.8)
IV	29 (27.9)
V	5 (4.8)
**BMI,** n (%)	
Normal weight (19> or <25)	30 (28.8)
Overweight (25> or <30)	41 (39.4)
Type I obesity (30> or <40)	28 (26.9)
Morbid obesity (>40)	5 (4.8)
**Level of Education, n (%)**	
Illiterate	3 (2.9)
Incomplete Elementary School	24 (23.1)
Completed elementary education	11 (10.6)
Incomplete high school	5 (4.8)
Completed high school	32 (30.8)
Incomplete higher education	4 (3.8)
Incomplete higher education	25 (24.0)
**Smoking**, n (%)	
Never	73 (70.2)
Discontinued for more than 6 months	24 (23.1)
Interrupted in the last 6 months	1 (1.0)
Currently makes use	6 (5.8)
**Alcoholism**, n (%)	
Never	66 (63.5)
Interrupted for more than 06 months	28 (26.9)
Interrupted in the last 06 months	1 (1.0)
Currently makes use	09 (8.7)
**Comorbidities (yes)**, n (%)	
Diabetes	16 (15.4)
Hypothyroidism	2 (1.9)
Skin disease	5 (4.8)
**Medicines (yes)**, n (%)	
Use of statins	16 (15.4)
Concomitant hormone therapy	61 (58.7)

Note: SD: Standard deviation; BMI: Body mass index; cm: centimeter.

All patients were diagnosed with Malignant Breast Neoplasm (ICD: C50), with a predominance of stage IIIA (27.9%), with Infiltrating Ductal Carcinoma (IDC) being the most frequent histopathological type (79.8%). Regarding treatment, most of the participants underwent previous chemotherapy or radical or conservative surgery, 61 participants (58.7%) were undergoing hormone therapy concomitantly with radiotherapy, 54 (51.9%) had their right breast irradiated, and 70 (67.3%) had the supraclavicular, frontal, axillary and inframammary fold regions irradiated.

Patients received, on average, 16 sessions of HFRT (SD = 1.5), with therapeutic protocols ranging from 15 to 20 sessions, with a total dose between 39.75 and 53.20 Gy (mean = 43.4; SD = 3.9) and fractionated dose between 2.50 and 2.90 Gy (mean = 2.7; SD = 0.4). Regarding the type of energy, 52.9% received 6 and 10 MV of photons and 80.8% did not receive electrons in the BOOST phase.

As for radiodermatitis, 76 (73.1%, 95%CI: 64–82) patients developed some degree during radiotherapy with first occurrence with mean dose of 35.4 Gy, with mean time of first occurrence in 13 days. Among the patients, 12 (11.5%) developed more than one level of radiodermatitis and three (2.9%) developed erythema, dry and moist desquamation during treatment. As for the levels of radiodermatitis developed during HFRT, the patients presented: erythema (72.1%, 95%CI: 63–81), dry desquamation (9.6%, 95%CI: 04–15) and moist desquamation (5, 8%, 95%CI: 01–10), as shown in Table 2. Regarding the most frequent development of radiodermatitis according to dose, fraction and irradiated area, 66 patients (63.5%) developed erythema in the axillary region with a mean dose of 36.5 Gy, seven (6.7%) had dry desquamation in the frontal region with a mean dose of 43.2 Gy and five (4.8%) had moist desquamation in the inframammary fold with a mean dose of 43.3 Gy. It is noteworthy that there was no occurrence of dry and moist desquamation in the supraclavicular region.

**Table 2.** Occurrence of erythema, dry and moist desquamation according to dose and irradiated area during treatment (n = 104). Brazil, 2021.

Outcomes	Occurrence during treatment		Occurrence per cumulative dose
	n (%)	95% CI	Mean (SD)
**RD incidence**	**76 (73.1)**	**64 – 82**	**35.4 (8.2)**
**Erythema**	**75 (72.1)**	**63 – 81**	**35.4 (8.3)**
Supraclavicular	30 (28.8)	20 – 38	40.6 (7.0)
Axillary	66 (63.5)	54 – 73	36.5 (7.9)
Frontal	53 (51.0)	41 – 61	37.4 (7.4)
Inframammary fold	45 (43.3)	34 – 53	38.7 (6.6)
**Dry desquamation**	**10 (9.6)**	**04 – 15**	**42.9 (4.1)**
Axillary	4 (3.8)	00 – 08	43.3 (3.9)
Frontal	7 (6.7)	02 – 12	43.2 (4.0)
Inframammary fold	2 (1.9)	–01 – 05	43.3 (3.9)
**Moist desquamation**	**6 (5.8)**	**01 – 10**	**43.3 (3.9)**
Axillary	2 (1.9)	–01 – 05	43.3 (3.9)
Frontal	2 (1.9)	–01 – 05	43.3 (3.9)
Inframammary fold	5 (4.8)	01 – 09	43.3 (3.9)

Note: RD: Radiodermatitis; CI: Confidence Interval; SD: Standard deviation.

Among the overweight patients, 48 (64.9%) developed erythema in the axillary region and 34 (45.9%) in the inframammary fold. Of the two patients who developed moist desquamation in the axillary region, all (100%) were overweight and of the five who developed moist desquamation in the inframammary fold, four (80%) were overweight. Among the six presenting dry desquamation, four developed it in the frontal region (66.7%) and two in the axillary region (33.3%). Of the 61 patients (58.6%) with chest circumference greater than 98 cm, who had voluminous breasts, 44 (72.1%) developed erythema, 10 (16.4%) developed dry desquamation and five (7.6%) developed moist desquamation.

In the present study, among the 61 (58.6%) patients who underwent concomitant hormone therapy to HFRT, 43 (70.5%) developed some outcome, with the development of erythema being the most frequent, namely: 42 (68.9%) had erythema, being more frequent in the axillary (63.9%) and frontal regions. (45.9%). Among the 10 patients who developed dry desquamation, seven (70%) used hormone therapy, and five (71.4%) developed dry desquamation in the frontal region. Among the five patients who developed moist desquamation in the inframammary fold, three (60%) were on concomitant hormone therapy.

Other results regarding the factors associated with a greater or lesser chance of developing radiodermatitis during HFRT are described in Table 3. It is observed that the use of statins significantly increases the chance of moist desquamation (OR = 1.1; 95% CI 1.01–1.13) and the breast volume significantly increases the chance of occurrence of dry desquamation (OR = 1.2; 95% CI 1.07–1.33). Having light brown skin, according to the Phototype III classification on the Fitzpatrick Scale, seems to be a protective factor for the occurrence of erythema (OR = 0.3; 95% CI 0.13–0.84).

**Table 3.** Factors associated with the development of acute radiodermatitis (n = 104). Brazil, 2021.

Associated factors	n (%)	Erythema	Dry desquamation	Moist desquamation
		OR (CI 95%)	OR (CI 95%)	OR (CI 95%)
**BMI >25**	74 (71.2)	1.8(0.72 – 4.48)	0.6(0.15 – 2.19)	2.1(0.23 – 18.78)
**Voluminous breast**	61 (58.7)	1.0(0.42 – 2.39)	**1.2(1.07 – 1.333)**	3.7(0.42 – 33.30)
**Phototype III**	56 (53.8)	**0.3(0.13 – 0.84)**	1.3(0.35 – 4.98)	1.8 (0.31 – 10.11)
**Concomitant hormone therapy**	61 (58.7)	1.5(0.61 – 3.64)	0.6(0.14 – 2.37)	0.7(0.12 – 3.97)
**Statins**	16 (15.4)	1.7(0.55 – 5.18)	1.7(0.20 – 14.50)	**1.1(1.01 – 1.13)**

Note: OR: Odds Ratio; CI: Confidence Interval; SD: Standard deviation; BMI: Body mass index.

**Figure 1.**
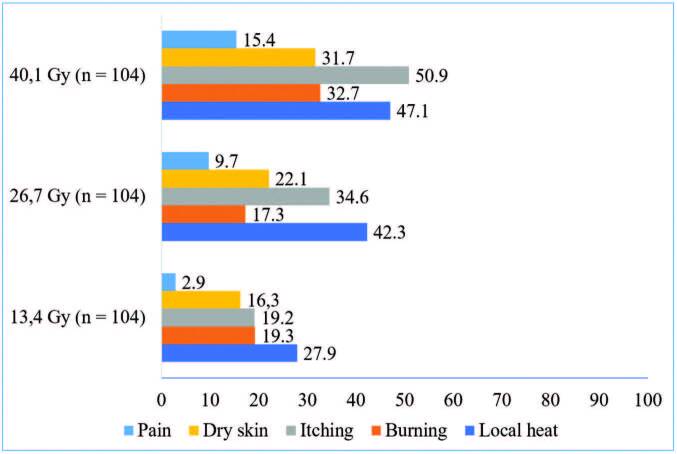
Representative graph of symptoms according to mean dose in Grays. Brazil, 2021.

As for the grading of the patients’ symptoms, weekly assessments were carried out and the results were explained according to the average dose each week in Figure 1. It should be noted that local heat (59.6%) and itching (59.6%) were the symptoms most reported by patients during treatment and were the symptoms most reported by patients for all doses of radiotherapy.

It is worth mentioning that in the average dose of 53.2 Gy, there was a variation in the sample size, since at the dose described, 103 patients had already completed radiotherapy.

## DISCUSSION

Previous studies have shown that the incidence of acute radiodermatitis during conventional radiotherapy ranges from 70 to 98%^3,9,10,16^. A systematic review with meta-analysis showed that HFRT significantly decreased severe acute skin reactions when compared to conventional RT (p = 0.02)^(17)^. The incidence of acute radiodermatitis in breast cancer patients is estimated to be dose-related^(10)^; however, no study estimates the incidence of acute radiodermatitis in breast cancer patients by relating the dose to the respective areas of the breast irradiated in HFRT, which is why the present study is unprecedented.

The incidence of radiodermatitis in breast cancer patients during HFRT was significant (73.1%), with erythema being the most frequent outcome, followed by dry and moist desquamation. The occurrence of erythema was more frequent in the axillary region, dry desquamation in the frontal region, and moist desquamation in the inframammary fold region. In breast cancer patients, delimiting radiodermatitis by the irradiated area is important, as there are often fold areas, such as the inframammary region and the axillary region, which favor the presence of moisture and friction, factors that can contribute to increased occurrence of radiodermatitis. Furthermore, knowing the incidence by areas of the irradiated breast may favor the standardization of protocols for better distribution and specificity of the dose provided by the radiation beams. Similarly, delimiting the dose at the beginning of the development of some degree of radiodermatitis favors intervention and early management in a specific way according to the characteristics of the breast region and its surroundings, which are very diversified. In this study, it was found that the occurrence of some degree of radiodermatitis occurred with an average dose equal to or greater than 36.5 Gy, which allows inferring that in HFRT the occurrence of radiodermatitis occurs with greater dose accumulation compared to the conventional radiotherapy.

In a study evaluating 392 patients with breast cancer, they concluded that race and ethnicity are not factors that predispose to severe toxicity, although black patients had a higher rate of moist desquamation (28%) when compared to white and brown (19%) patients^(18)^. One study observed, in a sample of 125 patients, that women who had fair skin had greater skin toxicity, such as widespread moist desquamation^(19)^. In the present study, we identified that patients with phototype III, which corresponds to light brown skin, with normal sensitivity to the sun and who burns moderately, have lower chance of developing erythema. For the other phototypes, there was no association between skin color and the development of some degree of radiodermatitis.

Previous studies have identified that BMI and breast volume are related to the development of radiodermatitis, because obese people or people with voluminous breasts commonly have larger regions of folds, making them susceptible to greater friction and, consequently, a greater chance of developing lesions^(10,20,21)^. In this study, 71.15% of the women had a BMI greater than 25, classified as obese or overweight. There was a higher incidence of erythema in the axillary regions and in the inframammary fold, of dry desquamation in the axillary region, and of moist desquamation in the axillary region and the inframammary fold related to overweight patients, as well as higher incidence of radiodermatitis grades in patients with voluminous breasts, which reinforces the evidence already found in the literature, which shows that larger breast volume is predictive for the development of radiodermatitis.

As for tobacco consumption, it was identified as a factor associated with severe skin reactions, as chronic exposure to tobacco hinders the skin healing process and causes changes in the physiology of systems, including the skin^(22,23)^. Among the patients evaluated in this study who reported current tobacco use or who had stopped using it in the last six months, all developed erythema, which is one of the initial signs of radiodermatitis.

Despite not being much described in the literature, a study^(24)^ demonstrated an association between the continuous use of statins and the development of radiodermatitis in the breast, increasing the chance of this radiotoxicity in grades II to IV by four times (erythema, dry and moist desquamation and ulceration). The same study reported that patients with hypothyroidism are more likely to develop radiodermatitis in the supraclavicular region earlier. In this study, we identified that among the patients who used statins, the majority developed erythema, with the frontal region being the most frequent. It was also found that patients who used statins are more likely to develop more severe signs of radiodermatitis. Although there was no association between hypothyroidism and supraclavicular toxicity, it should be noted that patients with hypothyroidism presented erythema in the frontal and axillary regions.

Regarding treatment-related factors, studies show that the use of oral hormone therapy increases the chance of developing acute radiodermatitis^(25,26)^. Patients on aromatase inhibitors developed moderate to severe erythema more often than those not on hormone therapy^(16)^. In the present study, although there was no significant association, there was a higher occurrence of acute radiodermatitis in patients who used hormone therapy concomitantly with radiotherapy, with erythema in the axillary and frontal regions and dry desquamation in the frontal region, which reinforces the evidence that oral hormone therapy is a factor associated with a greater chance of developing acute radiodermatitis.

Regarding dose accumulation and the development of radiodermatitis, in previous studies^(10,17,27)^, HFRT (average 42.5 Gy in 16 fractions) was responsible for the occurrence of equal or lesser cutaneous toxicity than the fractionation of conventional radiotherapy. A study carried out in the same service of this study between March 2016 and May 2017 showed that the time for erythema to occur in breast cancer patients undergoing radiotherapy was, on average, 11 days, being present mainly in the third and fourth weeks of follow-up. Dry desquamation occurred between the fourth and fifth weeks of treatment and moist desquamation started in the third week and had an increase in occurrence in subsequent weeks^(10)^.

Thus, as radiodermatitis is dose-dependent, symptoms such as itching, heat and dry skin are commonly related to its development, as described in the literature^(15)^. As observed in this study, as dose accumulation increased, so did reports of typical symptoms over the course of treatment. In general, the appearance of erythema is related to the accumulation of a mean dose of 35.4 Gy, which may be associated with the increase in reports of local heat, burning, itching and dry skin between the third and fourth evaluation, as they are characteristics related to the development of erythema. The appearance of dry desquamation occurred with a mean dose of 42.9 Gy, which may be related to the increase in reports of itching and burning reported between the fourth and fifth evaluations. Reports of pain, a symptom related to the appearance of moist desquamation that developed with a mean dose of 43.3 Gy, were also reported by 16 patients (15.4%) in the fourth evaluation and by one patient (100%) in the fifth evaluation.

Radiodermatitis in more severe grades can cause delays in treatment or even interruption of radiotherapy, preventing therapeutic success and causing discomfort, pain and decreased quality of life for the patient^(2,28,29)^. In addition, the interruption of a patient’s treatment, in addition to jeopardizing the success of the therapy, causes a delay in starting another treatment, additionally leading to more expenses for the service^(10)^.

It is also important to highlight that the recommendations on skin care in general are simple and following the protocols guided by the nurse in the department can delay the time of occurrence and the severity of radiodermatitis. Clear and objective patient orientation and the use of educational manuals, technologies such as games or applications aimed at self-care^(13,30)^, as well as telenursing are easy and accessible technologies to assist in the management of toxicities, so that information can be transferred to the patient and extended to family members who are important support networks for the success of the treatment. Furthermore, photographic recording is also an important tool to assess the development of the grade of radiodermatitis throughout the treatment, allowing monitoring of the development and area of extension of acute radiodermatitis.

## LIMITATIONS OF THE STUDY

One of the limitations of the study is due to the constant technical problems and maintenance in the treatment devices model CLINAC CX and model PRIMUS. This, coupled with the fact that the responsible company not having a branch in Brasília and the parts being expensive, caused problem-solving delay and certain days with the device not working. Device operation was interrupted for 1 to 2 days during the treatment of 49 patients included in this study. It should be noted that this complication may have contributed to the underreporting of the occurrence of radiodermatitis, especially with regard to its severity.

Moreover, one of the biggest limitations of the study was the emergence of the COVID-19 pandemic, caused by the new coronavirus, called SARS-CoV-2. The pandemic hindered patients follow-up, the frequency of nursing consultations, and consequently, skin assessment with the photos in the previously established time and the continuity of treatment, which was occasionally interrupted due to flu-like symptoms reported by patients.

## CONTRIBUTIONS TO THE NURSING AND HEALTH AREA

Considering the decrease in the incidence of acute radiodermatitis in patients treated with HFRT when compared to treatment with conventional radiotherapy, this study highlights that the use of hypofractionated radiotherapy implies lower occurrence of cutaneous toxicities during treatment.

However, even with HFRT, a high incidence of acute radiodermatitis is observed in breast cancer patients. Thus, the study reinforces the importance of developing a more individualized treatment plan, with the use of more specific doses for each patient, according to the irradiated area, taking the factors associated with a greater chance of developing radiodermatitis, such as voluminous breasts and use of statins, into account.

In addition, it is extremely important to reinforce self-care and patient participation during treatment, through the performance of essential skin care and compliance with the protocols institutionalized by each service, through educational manuals, applications or technologies aimed at self-care^(13,30)^, thus favoring not only the reduction of the most severe grades of radiodermatitis, but the dissemination of knowledge to caregivers and family members. The results of this study encourage the development of technologies aimed at self-care with the inclusion of risk factors related to a greater chance of developing radiodermatitis, care for the prevention of these factors and care aimed at preventing the development of radiodermatitis, especially in skinfold regions.

## CONCLUSIONS

The incidence of radiodermatitis in patients with breast cancer was 73.1%, with erythema being more frequent in the axillary region. The average dose for the occurrence of erythema was 35.4 Gy, for dry desquamation was 42.9 Gy and for moist desquamation, 43.34 Gy. The most reported symptoms by patients during treatment were local heat and itching. With regard to associated factors, voluminous breasts and the use of statins increase the chances of developing more severe degrees of radiodermatitis. However, light brown skin (phototype III) seems to be a protective factor for the occurrence of erythema. The results found reinforce the importance of developing care protocols for the management of radiodermatitis in breast cancer patients who underwent HFRT.
